# Cancer of the Stomach

**Published:** 1844-12-28

**Authors:** 


### CANCER OF THE STOMACH.

According to Professor Rostan, this denomination is improper, applied as it is to different lesions, such as encephaloides, scirrhus, &c., an opinion which has already been expressed by Professor Andral, and many other authors. The disease does not, as is generally stated, commence with hypertrophy; the membranes not only increase in volume, but likewise are in a diseased state, which does not take place in hypertrophy, where the parts are simply thicker than usual. In cancer, they are generally changed into an amorphous mass, which presents none of the characters of the constituent parts of the organ. On other occasions, on dividing the mucous membrane, it is found simply hypertrophied, and, on penetrating deeper, the fibrous and muscular coats are perceived in a similar condition. When the thickening has reached a certain degree, the parts assume an appearance like bacon; they become firmer, and produce, when cut, the crackling noise characteristic of scirrhus. This degenerescence may not only affect the stomach, but also extend to the surrounding organs, even the vertebral column. As to the frequency of the disease in the different parts of the stomach, they may be classed as follows: pylorus, cardia, little curvature, great curvature, and, lastly, the whole organ at the same time.

The alteration may affect:—1° *The vessels*, causing hæmorrhages; this accident may, however, not only be produced by ulceration, but likewise by exhalation; 2° *The nerves:*—Dr. Prus, who has published an important work on the state of the pneumogastric nerve in cancer of the stomach, states that, frequently, it is impossible to distinguish it from the diseased parts. The three principal signs of this affection are, vomiting of a dark liquid, pain and swelling; they may, however, exist without cancer, and *vice versa.* The following cases are proofs. A young woman entered the *Salpetriere*, presenting a tumour in the epigastrium, accompanied by vomiting and pain, and after remaining some time in the hospital, left it much in the same state as on her entry. Many years after, having met with the same person, I was astonished to find that the tumour had completely disappeared, and with it the other morbid symptoms. At her death the autopsy having been performed, I found the gastric mucous membrane healthy, and that the tumour was formed by a cyst situated between the rectus and transversalis muscles, nothing remaining but a small kernel, the liquid portion having been absorbed. A woman, in the same hospital, had experienced, when 30, all the characteristic symptoms of cancer of the stomach; the vomiting after some time disappeared, and the patient lived, or rather languished, twenty years longer. At her demise, the cessation of the emesis was explained by the existence of an ulceration, which had enlarged the pylorus, previously narrowed by the cancer. But this last mentioned symptom may, as Dr. Magendie has demonstrated, be owing to the contraction of the abdominal muscles, and it is thus that we may account for persons, the coats of whose stomachs are incapable of contracting, being still able to reject the substances contained in that organ. An important question here presents itself. Can this disease be cured? The professor answers affirmatively, and quotes in favour of this opinion several cases: that of Beclard, who died of an affection of the brain, after having presented, some years previous to that event, evident signs of an organic disease of the stomach. At the *post mortem* examination a cicatrix was found on the mucous membrane of the stomach. Also, that of a young rachitic woman, who offered all the characteristic signs just described: vomiting; pain; tumour in the epigastrium; yellow tint of the skin; all of which, however, disappeared, and the patient died many years after of disease of the heart, on examining the stomach, the mucous membrane, lining the pylorus, was wrinkled by the seat of a fan-like cicatrix, indicating manifestly, the previous existence of an ulceration. It must not be forgotten that tumours, in elderly persons, may be formed by a collection of fæculent matter in the transverse colon, and be accompanied by pain and vomiting; in this case, a purgative will set all to rights.—*Ibid, from Gaz. des Hop.*

